# Electroencephalographic Correlates of Sensorimotor Integration and Embodiment during the Appreciation of Virtual Architectural Environments

**DOI:** 10.3389/fpsyg.2015.01944

**Published:** 2015-12-22

**Authors:** Giovanni Vecchiato, Gaetano Tieri, Andrea Jelic, Federico De Matteis, Anton G. Maglione, Fabio Babiloni

**Affiliations:** ^1^Department of Physiology and Pharmacology, Sapienza University of RomeRome, Italy; ^2^Laboratory of Social Neuroscience, IRCCS Fondazione Santa LuciaRome, Italy; ^3^Department of Psychology, Sapienza University of RomeRome, Italy; ^4^Department of Architecture and Design, Sapienza University of RomeRome, Italy; ^5^Department of Molecular Medicine, Sapienza University of RomeRome, Italy

**Keywords:** electroencephalography, immersive virtual reality, presence, architecture, embodiment, sensorimotor integration, spatial navigation, affordances

## Abstract

Nowadays there is the hope that neuroscientific findings will contribute to the improvement of building design in order to create environments which satisfy man's demands. This can be achieved through the understanding of neurophysiological correlates of architectural perception. To this aim, the electroencephalographic (EEG) signals of 12 healthy subjects were recorded during the perception of three immersive virtual reality environments (VEs). Afterwards, participants were asked to describe their experience in terms of Familiarity, Novelty, Comfort, Pleasantness, Arousal, and Presence using a rating scale from 1 to 9. These perceptual dimensions are hypothesized to influence the pattern of cerebral spectral activity, while Presence is used to assess the realism of the virtual stimulation. Hence, the collected scores were used to analyze the Power Spectral Density (PSD) of the EEG for each behavioral dimension in the theta, alpha and mu bands by means of time-frequency analysis and topographic statistical maps. Analysis of Presence resulted in the activation of the frontal-midline theta, indicating the involvement of sensorimotor integration mechanisms when subjects expressed to feel more present in the VEs. Similar patterns also characterized the experience of familiar and comfortable VEs. In addition, pleasant VEs increased the theta power across visuomotor circuits and activated the alpha band in areas devoted to visuospatial exploration and processing of categorical spatial relations. Finally, the de-synchronization of the mu rhythm described the perception of pleasant and comfortable VEs, showing the involvement of left motor areas and embodied mechanisms for environment appreciation. Overall, these results show the possibility to measure EEG correlates of architectural perception involving the cerebral circuits of sensorimotor integration, spatial navigation, and embodiment. These observations can help testing architectural hypotheses in order to design environments matching the changing needs of humans.

## Introduction

Despite increasing evidence of the influence physical features in the built environments have on our psychophysiological states (Stamps, 1999; Lindal and Hartig, 2013), systematic research on the cerebral networks activated by perception and appreciation of architectural spaces is still scarce. At the same time, there is a growing trend in architectural practice of employing the evidence-based insights for creating environments capable of satisfying the need for variety and improving people's psychological, biological and social lives. In this regard, the present study aims to illustrate the potential that neuroscientific findings have for describing the impact of architecture on people.

First of all, buildings have to respond to various functional requirements such as adequate lighting, heating, and cooling systems as well as public safety provisions. These functional aspects of architectural design are strongly supported by contemporary technological advances. However, the understanding of how the aesthetic perception of living environments affects people's cognitive and emotional states primarily relies on the architect's intuition and experience. In this context, available research provides good indications that personal sensory motor perception of the environment could play an important role in cognitive and emotional interactions within the environment itself. Most importantly, these attributes can now be evaluated from the neurophysiological point of view. Therefore, it becomes relevant to understand the human cerebral reactions produced by the perception of architecture. This claim is generally supported by different studies from various disciplines, including environmental psychology, behavioral research, and biophilic design.

More specifically, Appleton's habitat theory (Appleton, 1996) states that considering an environment as emotionally and aesthetically pleasing is indicative of its favorability to survival. In fact, since spatial features influence the activities and social interactions to be performed in a specific environment (i.e., sleeping in a bedroom or entertaining guests in a living room), architecture can affect cognitive and emotional states of inhabitants as well as their mood and productivity (Graham et al., 2015). These authors take into account different perceptual dimensions, highlighting the role of comfort as an important factor in both home and work environments in order to promote well-being and productivity. Additionally, architectural design can either limit or facilitate the social interactions and the dynamics that take place at home (Graham et al., 2015). Furthermore, studies show that the perception of different kinds of environments can have a beneficial impact on the observer's cognitive ability and task performance. For instance, the exploration of an environment can promote long-term potentiation in the hippocampus, improving memory encoding.

Although direct evidence of a link between exploration of environments and increased plasticity comes from studies on animals, such a link has been also found in humans. Indeed, an improvement of the recall of both allocentric spatial information (Plancher et al., 2013) and words (Schomaker et al., 2014), as well as memory encoding (Bunzeck and Düzel, 2006) was demonstrated when familiar items were presented during the active exploration of new environments.

In addition, evidence of the cerebral responses underpinning perceptual dimensions such as pleasantness is also found in studies on art appreciation, showing the activation of the motor system through embodied mechanisms, encompassing the simulation of actions, emotions, and corporeal sensations, which are also postulated to play an important role in the perception of architecture (Freedberg and Gallese, 2007). The idea of the involvement of non-overt bodily reactions in the perception and the experience of architectural spaces can be traced back from late nineteenth century “Einfühlung” theories. Such hypotheses suggest that the observation of architectural forms may lead to corporeal responses establishing a relationship between the aesthetic and emotional dimension, as well as bodily engagement with space (Mallgrave and Ikonomou, 1994). These assumptions have been validated by recent neuroscientific findings, highlighting the crucial role of sensorimotor areas in the appreciation of works of art (Kawabata and Zeki, 2004; Umilta' et al., 2012; Sbriscia-Fioretti et al., 2013). Freedberg and Gallese (2007) proposed a theoretical framework based on the role of embodied simulation and empathy in the aesthetic experience of art resulting in tactile sensations, implied gestures and actions. Additionally, the interaction with the built environment as well as its appreciation could also involve motivational and affective factors. In fact, the perception of visual artwork (Sbriscia-Fioretti et al., 2013) and environments characterized by curvilinear contours (Vartanian et al., 2013) activates reward circuits formed by medial orbitofrontal and anterior cingulate cortices (Vartanian and Goel, 2004). Moreover, observation of art or architecture may be accompanied by activations of neural networks regulating reward and judgment, suggesting the involvement of emotional, cognitive and contextual factors mediating aesthetic appreciation (Chatterjee and Vartanian, 2014).

However, very few neuroscientific studies have been conducted so far that investigate the modulation of the cerebral activity during perception of real-like architectural environments. One of the reasons for this lack of knowledge consists in a methodological gap. Concretely, there is a difficulty in reproducing the qualitative richness of architectural spaces in highly controlled environments such as a laboratory, allowing systematic neurophysiological investigation. However, a growing number of studies in psychology and neuroscience demonstrate that such difficulties can be surmounted using the Immersive Virtual Reality (IVR; Sanchez-Vives and Slater, 2005; Bohil et al., 2011). The IVR technologies create a high sensorial immersion within a fictive three dimensional scenario, inducing in the observer the sense of presence defined as the “sense of being in the virtual environment” (Slater and Wilbur, 1997) that is, a psychophysiological state which reproduces realistic behaviors and physiological responses as if the subject was experiencing a real-life situation (Diemer et al., 2015). Hence, by using the IVR it is possible to create highly controlled real-size architectural environments, allowing the measurement of reliable behavioral and neurophysiological indices.

The objective of this study is to investigate whether simple architectural scenarios are able to modify perception as well as to explore its corresponding cerebral activity. An IVR paradigm was used to highlight the neurophysiological features underpinning architectural perception by analyzing the electroencephalographic (EEG) and autonomic reactions elicited by the perception of highly immersive real-size Virtual Environments (VEs). With the working hypothesis that changing interior design features could activate the cerebral circuits involved in mechanisms of embodiment in different ways, we compared the spectral activity of the EEG using a set of subjective dimensions describing the perception of VEs such as Pleasantness, Novelty, Familiarity, Comfort, Arousal, and Presence. In addition, the hypothesis that architectural perception could involve cerebral circuits regulating reward and affective processes was also advanced basing on a broad literature on aesthetic judgments (for a review, see Chatterjee and Vartanian, 2014).

## Materials and methods

### Participants

Twelve healthy volunteers were involved in the study (five females; mean ± SD 26.8 ± 2.4). All subjects had normal or corrected-to-normal vision and were not familiar with the IVR experience. The study was approved by the ethics committee of Fondazione Santa Lucia, according to the ethical standards of the 1964 Declaration of Helsinki.

### Procedure

**Figure 1:**
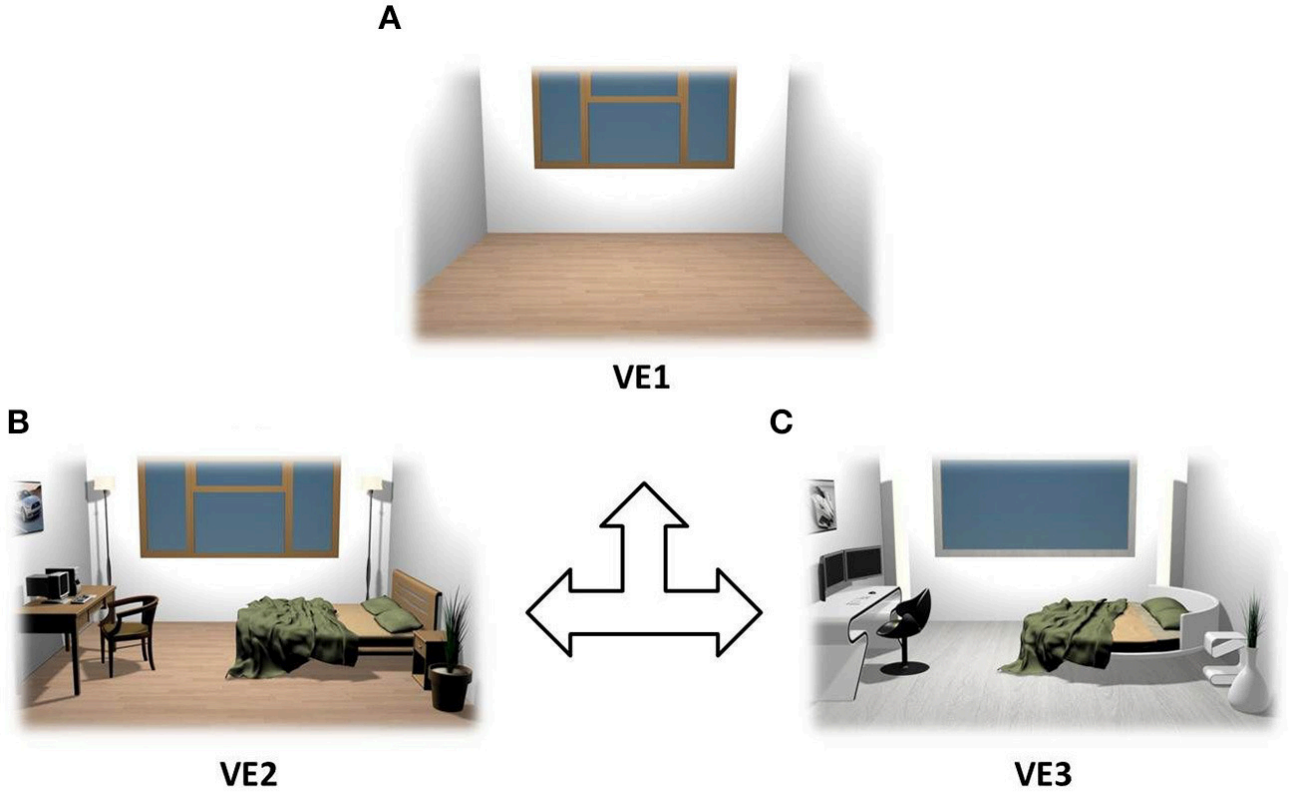
**Rendering of the VEs that were used as stimuli**. VEs were counterbalanced across subjects: empty room **(A)**, modern furniture **(B)**, and cutting edge design **(C)**.

Three VEs were designed in real size (5 × 5 m) and tested using different interior designs. The first one represented an empty room (VE1), which was in turn equipped with a modern design (VE2) and then with cutting-edge furniture (VE3) as shown in Figure 1. They were designed using 3DS Max 2011 (Autodesk, Inc.) and implemented in XVR (http://www.vrmedia.it/en/xvr.html; Tecchia et al., 2010).

**Figure 2:**
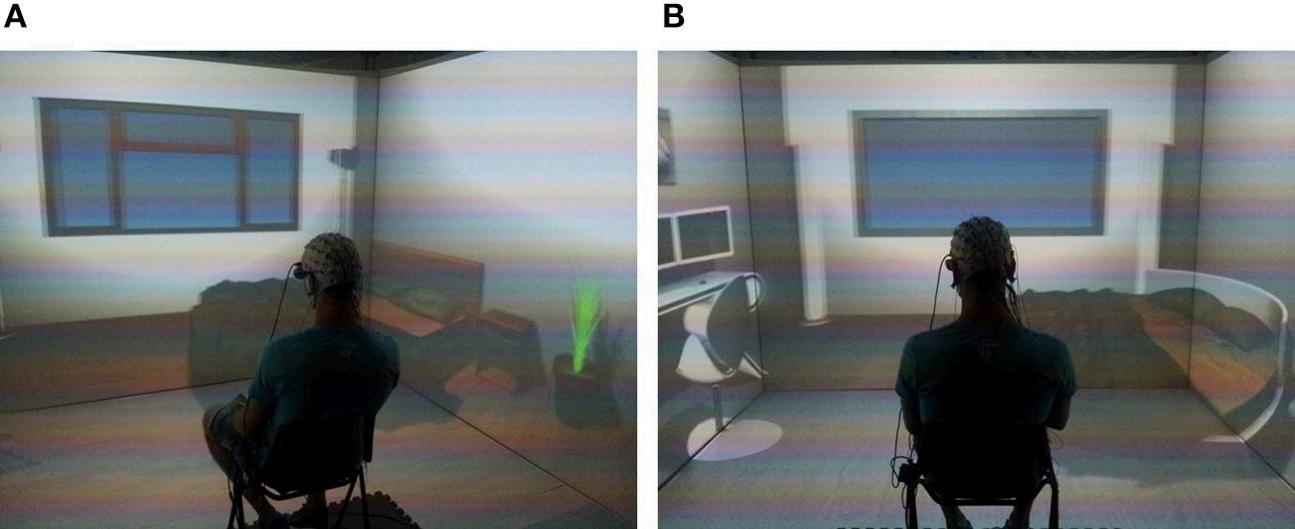
**Virtual reality cave system setup**. It is possible to appreciate two rooms with different interior design: modern furniture **(A)** and cutting edge design **(B)**. The subject is placed in the middle of the cave, wearing the EEG cap, for the whole duration of the experiment.

The VEs were presented by means of a CAVE automatic virtual environment system (3 × 3 × 2.5 m; Cruz-Neira et al., 1993) composed by three rear-projection screens for the walls and a down- projection screen for the floor, as illustrated in Figure 2. Viewsonic projectors (1024 × 768 pixels) displayed images on the screens through mirrors and an Nvidia 3d vision wireless glass provided the image of 3D graphics generated by the CAVE. An Intersense 900 ultrasonic head tracking system (6° of Freedom, DoF) was used to record in real time the subject's head position and orientation (with a frequency of 120 Hz) and thus to anchor the 3D images to his/her point of view. As a special feature of the CAVE system, the four 3D images were joined together so that subjects could not see the edges of the adjacent walls. Hence, the active stereo projection was perceived as a continuous virtual world. This setup enabled a high level of sensorial immersion so that the subject could feel herself/himself physically present in the VE (Sanchez-Vives and Slater, 2005).

Subjects sat on a chair placed in the middle of the CAVE and immersed in each VE for 4 min. VEs presentation was counterbalanced among subjects. Their task was (i) to visually explore the surrounding environment and, at the end of the exposure, (ii) to verbally express their judgment about the perceptual dimensions of Familiarity, Novelty, Comfort, Pleasantness, Arousal, and Presence using a 9-point rating scale (1, lowest score; 9, highest score). These items were arranged in two short questionnaires, as shown in Table 1, each related to perception and sense of presence induced by the VEs, respectively. The items used to measure the sense of Presence were adapted from Sanchez-Vives and Slater (Sanchez-Vives and Slater, 2005). Subjects had 2 min to answer the questionnaires before experiencing the next VE. The whole procedure lasted around 20 min during which the EEG and autonomic activity were continuously collected, as described in the following section.

**Table 1:** **Items used to assess dimensions of perception and presence**.

	**Dimension**	**Item**
Perception	Familiarity	How much did this VE remind you of environments in which you lived?
	Novelty	How much did this VE provide elements of novelty with respect to environments in which you lived?
	Comfort	How much did you feel at your ease during the perception of this VE?
	Pleasantness	How much did you like this VE?
	Arousal	How much did this VE arouse you?
Presence		To what extent did you have the sense of being in the VE?
		To what extent were there times during the experience when the VE became the reality for you and you almost forgot about the real world of the laboratory in which the whole experience was really taking place?
		When you think about this experience, do you think of the VE more as images that you saw or more as somewhere that you visited?

*Each dimension was evaluated according to a 9-point scoring scale (1, lowest; 9, highest)*.

At the beginning, each subject experienced a familiarization period (Slater et al., 2010; Tieri et al., 2015a,b) within an additional neutral VE that represented a generic laboratory setting composed by different objects including a chair, a desk, some computers, and books. Subjects were asked to look around the environment and verbally describe the virtual scenario. This preliminary phase ended when they reported to feel present in the environment (all participants experienced presence within 81.28 ± 37.62 s). The neurophysiological activity acquired during this phase was not used for further analysis.

### Behavioral data analysis

With the aim to investigate the relationship among each of the perceptual dimensions—Familiarity, Novelty, Comfort, Pleasantness, Arousal—the subjective behavioral scores were transformed in z-scores and then used to perform Pearson's correlation analysis (Bonferroni corrected due to multiple tests). Afterwards, two datasets for each perceptual dimension were created using the z-scores in order to identify and group VEs that had the highest and lowest scores, respectively, according to each subject. Therefore, positive (*z* > 0) scores represented VEs which were highly rated (e.g., High Pleasantness VEs) while negative (*z* < 0) scores were associated with lowly rated VEs (e.g., Low Pleasantness VEs). These z-scores were used to contrast the neurophysiological data: interiors rated with positive scores (e.g., High Pleasantness) were compared with the ones rated with negative scores (e.g., Low Pleasantness) for each perceptual dimension and presence.

According to this procedure, the analysis was driven by the subjective scores across all VEs instead of investigating the cerebral activity related to the perception of single VEs (see Vecchiato et al., 2010a, 2011b for a similar statistical approach). The subjective scores related to Presence were first averaged across the three items and then z-scored across stimuli. These were first used to separate VEs that induced either high or low presence and, subsequently, to group and contrast the neurophysiological activity. The data associated to *z* = 0 were not considered for the following analysis (6.48% of total scores).

### Electroencephalographic and autonomic signal recording and processing

The EEG activity was recorded by means of a portable 24-channel system (BEmicro, EBneuro, Italy). Nineteen electrodes were disposed according to the 10–20 I.S. The signals acquired at a sampling rate of 256 Hz with sensors impedances kept below 10 kΩ. Raw EEG traces were band pass filtered (*hp* = 0.5 Hz; *lp* = 45 Hz) and the Independent Component Analysis (ICA; Hyvärinen and Oja, 2000) was then applied to detect and remove components generated by eye movements and blinks. For this purpose, the infomax ICA algorithm was used, provided by EEGLAB (Delorme and Makeig, 2004). EEG data were extracted to take into account the perception of the three VEs (three stimuli per subject) and further segmented into 1-s epochs (240 epochs per stimulus defining the 4 min exposition). Muscular and environmental artifacts were detected and removed with a semi-automatic procedure based on two different criteria: threshold (traces which exceeded a threshold of ±80 μV were rejected) and gradient (traces in which the difference between two consecutive samples exceeded ±50 μV were rejected). Only artifact-free trials (92.72%) were considered for this analysis. The extra-cerebrally referred EEG signals were transformed by means of the Common Average Reference (CAR). Afterwards, the Power Spectral Density (PSD) was computed for each epoch according to the Welch method (Welch, 1967) with Hanning window in a Matlab environment (The MathWorks, Inc.). Individual Alpha Frequency (IAF) was calculated for each subject to perform time-frequency analysis according to individually defined bands and widths (Doppelmayr et al., 1998). Therefore, in this study the bands of interest were defined as theta, ranging from IAF × 0.4 to IAF × 0.8 Hz, alpha (IAF × 0.8, IAF × 1.2) Hz and mu (IAF, IAF × 1.2) Hz.

In all subjects we achieved an IAF = 10.54 ± 0.80 Hz. Then, the PSD was band averaged to obtain data structures comprising *T* = 240 time-frequency bins per EEG channel and subject, for the three frequency bands. The whole dataset was pooled according to the behavioral z-scores in order to contrast the positive against the negatively judged VEs. This comparison was performed for each perceptual dimension (Familiarity, Novelty, Comfort, Pleasantness, Arousal) and Presence. Hence, a PSD time-frequency series was obtained for each condition.

Autonomic activity, such as Electrodermal Activity (EDA) and Heart Rate (HR), was recorded by means of a NeXus-4 (Mindmedia, The Netherlands) system with a sampling rate of 256 Hz. Skin conductance was acquired by the constant voltage method (0.5 V). Ag-AgCl electrodes were attached to the palmar side of the middle phalanges of the second and third fingers of the participant's non-dominant hand with a Velcro fastener, following published procedures (Boucsein et al., 2012). In order to retrieve the tonic component of the skin conductance (Skin Conductance Level, SCL) the LEDAlab software was employed (Benedek and Kaernbach, 2010). Disposable Ag-AgCl electrodes, which were provided by the Mindmedia company, were applied to the subject's wrist to collect cardiac activity. The Pan-Tompkins algorithm was then used to calculate the HR (Pan and Tompkins, 1985). Both EDA and HR were analyzed to asses Presence.

### PSD non-linear time-frequency analysis

In order to account for non-linear dynamics of the EEG elicited during the continuous perception of the VEs, a method was developed inspired by time-delay embedding procedures (Stam, 2005, 2006). The application of non-linear dynamics to electroencephalography, often referred to with the term “chaos theory” (Elbert et al., 1994), paved the way to a new perspective for the study of normal and disturbed brain function (Stam, 2003). In fact, non-linear dynamics studies of the EEG are applied in wide research domains ranging from resting to active mental states (Aftanas and Golocheikine, 2002; Tirsch et al., 2004, see Stam, 2005 for a review). The wide use of non-linear analysis of the EEG is justified by the fact that levels of synchronization of functional sources are not constant over time, but show peculiar fluctuations which have a scale-free character (Stam and de Bruin, 2004).

In this analysis, the *PSD* time-frequency bins were averaged according to a changing time-window depending on the values of its autocorrelation function. Specifically, in *x*[*n*] = *PSD*_*b, ch*_[*n*], *PSD* is the time series of spectral values defined for the frequency *b* and channel *ch*. For each increasing *t*_*N*_ time window of length *N*, with *N* ϵ [1, *T*], the autocorrelation function was computed as:

$$\begin{array}{l} R_{xx}\left[m\right]=R_{xx}\left[-m\right]=\overset{N}{\underset{n=1}{\sum }}x\left[n\right]x\left[n-m\right] \end{array}$$

with *m* ϵ [1-*N, N*-1]. Here are considered only values of *R*_*xx*_ with *m* ≥ *0*. At each iteration *t*_*N*_, *L*_*N*_ equals the length of the time window, after which the autocorrelation function of the time series dropped to 1/e of its maximum value (Stam, 2005). Then, the weighted average of the *PSD* for each *t*_*N*_ was computed as:

$$PSD_{w}\left[t_{N}\right]=\frac{\sum_{n\;=\;1}^{L_{N}}x\left[n\right]w\left[n\right]}{\sum_{n\;=\;1}^{L_{N}}w\left[n\right]}$$

where *w*_*n*_ are the selected *L*_*N*_ larger coefficients of the autocorrelation function. This calculation was performed for each subject and condition. Afterwards, the subjective z-score of *PSDw* spectral values for each time bin was calculated, as similarly done with behavioral scores, to perform the mass univariate analysis described in the following paragraph.

### Statistical mass univariate analysis

EEG features are typically analyzed via statistical methods on average activity in a priori windows. Mass univariate analyses were born thanks to the advances in computing power and statistics (Blair and Karniski, 1993). They consist of hundreds or thousands of statistical univariate tests, e.g., Student's *t*-test, which are applied to a large number of time points or cerebral locations accompanied by corrections for multiple comparisons. Such analyses are very useful when there is little a priori knowledge of effect locations or latencies, as well as to delineate effect boundaries. For instance, conducting statistical analysis on particular cerebral features such as average or peak amplitude does not take into account the whole time-series of observations and, therefore, cannot provide information about when and/or where an effect occurs (Dien and Santuzzi, 2005). This analysis can be applied to EEG, magnetoencephalographic (MEG), and functional Magnetic Resonance Imaging (fMRI) data, as reviewed in Groppe et al. (2011). Here, this methodology is applied to the z-scored *PSDw* time series in order to contrast the spectral activity for each perceptual dimension and presence. This is made using multiple Student's *t*-test (significance level of 0.05) and the False Discovery Rate (FDR) correction for multiple comparisons to minimize type I errors (Benjamini and Yekutieli, 2001; Vecchiato et al., 2010b).

In the following figures, statistical results provided by the mass univariate analysis are shown by means of raster diagrams. Average *t*-test results in specific time-windows are also summarized by means of scalp topographic maps.

## Results

### Behavioral results

**Figure 3:**
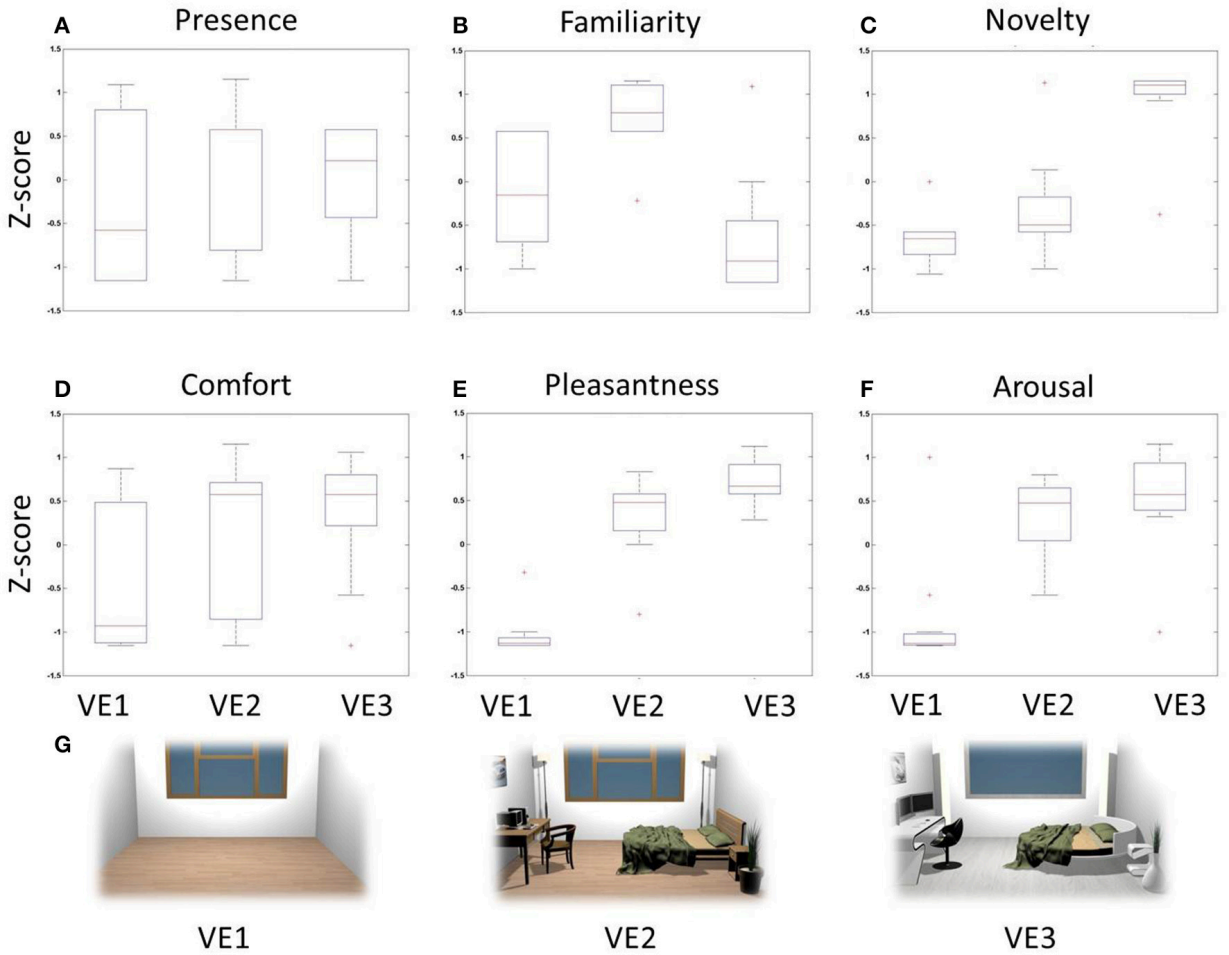
**Z-score boxplots of behavioral judgments among VEs**. Each panel contains three boxplots related to the distribution of behavioral scores for VE1 (left), VE2 (center), and VE3 (right). On each box, the central mark is the median, the edges of the box are the 25th and 75th percentiles, the whiskers extend to the most extreme data points. Red crosses indicate outliers of the distribution. **(A)** Presence, **(B)** Familiarity, **(C)** Novelty, **(D)** Comfort, **(E)** Pleasantness, **(F)** Arousal, **(G)** Two-dimensional rendering of the three VEs designed and used for the stimulation.

In Figure 3 there are several boxplots showing the z-score distributions of the judgments related to the adopted behavioral dimensions. These graphs show that the VE judged highly familiar was the one characterized by modern furniture, while novelty dimension returned highest scores for the one with cutting-edge furniture. Instead, scores related to pleasantness and arousal revealed that subjects assigned the highest scores to interiors with objects in general, indistinctly of the kind of furniture.

The degree of correlation between perceptual dimensions is shown through the computation of the Pearson's coefficients (Table 2). In particular, judgments of Novelty were positively correlated with Pleasantness (*R* = 0.68, *p* < 0.01) and negatively with Familiarity (*R* = −0.52, *p* < 0.01). Judgments of Pleasantness were also positively correlated with Arousal (*R* = 0.63, *p* < 0.01). These results illustrate that the measured perceptual dimensions are characterized by a certain degree of correlation, which was investigated through cerebral data.

**Table 2:** **Pearson's correlation coefficients among perceptual dimensions**.

	**Familiarity**	**Novelty**	**Comfort**	**Pleasantness**	**Arousal**	**Presence**
Familiarity	1	^*^−0.52	0.08	−0.06	−0.07	0.11
Novelty		1	0.27	^**^0.68	0.31	0.18
Comfort			1	0.43	0.19	0.39
Pleasantness				1	^**^0.63	0.21
Arousal					1	0.12
Presence						1

* *p < 0.05*,

** *p < 0.01, Bonferroni corrected for multiple comparisons*.

### PSD time-frequency pattern and autonomic variables of presence

The z-scores computed for all behavioral dimensions were used to pool the whole *PSDw* dataset into groups for the mass univariate analysis. First, the z-scores related to the Presence dimension were used in order to (i) identify the autonomic and EEG correlates of presence and (ii) to investigate possible time windows showing most activation during the whole 4 min perception of the VEs. To this aim, HR, SCL and *PSDw* related to positive z-scores of Presence were grouped in the High Presence dataset (19 observations) while the HR and *PSDw* related to negative z-scores formed the Low Presence dataset (13 observations).

**Figure 4:**
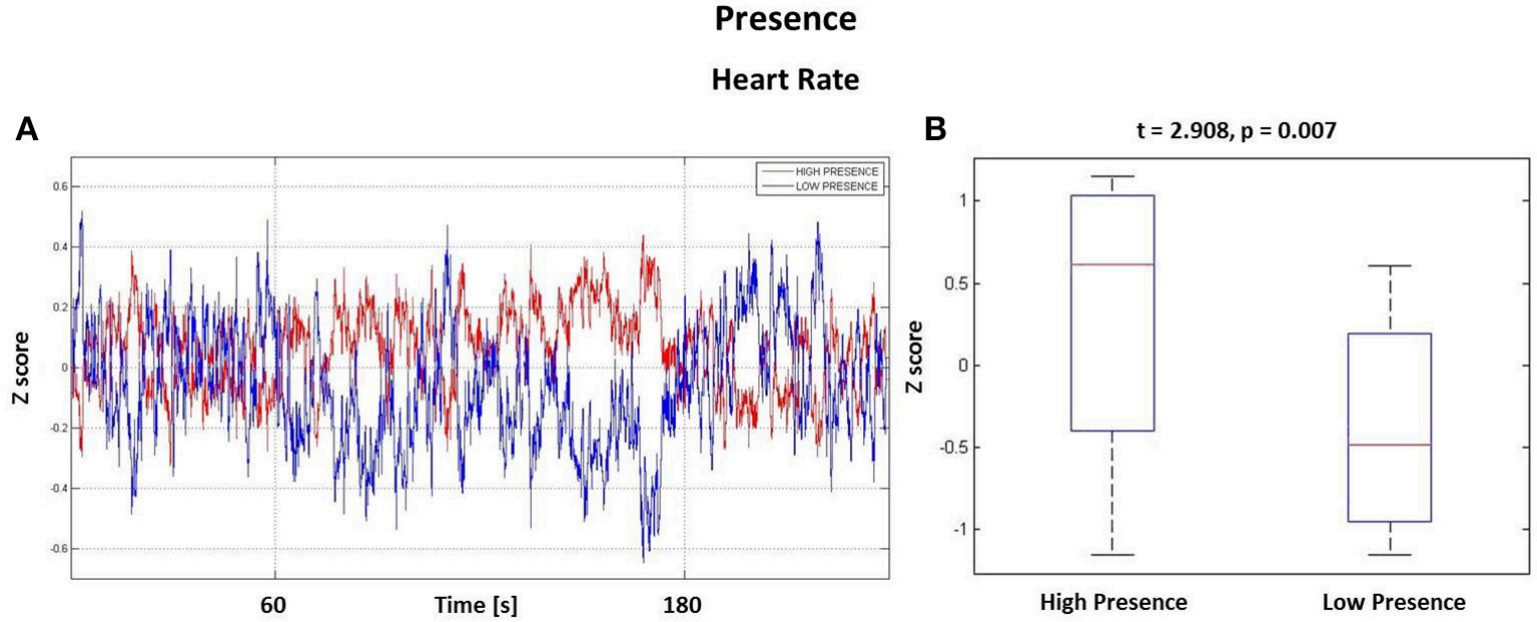
**Results from the Heart Rate analysis**. **(A)** HR waveforms for the High Presence (red) and Low Presence (blue) conditions. Vertical grid highlights the time window *t*_*m*_, the central part of experience, where the highest difference was found. **(B)** Boxplots showing the z-score distributions of High and Low Presence conditions computed in the time interval *t*_*m*_. On each box, the central mark is the median, the edges of the box are the 25th and 75th percentiles, the whiskers extend to the most extreme data points. Difference is significant with *t* = 2.908 and *p* = 0.007.

The HR waveforms were z-scored and averaged across subjects, as visible in Figure 4A. Here it is possible to appreciate that the largest difference between the two groups is within the time window *t*_*m*_ = [60, 180] s comprising minutes 2 and 3, i.e., the central part of the experience in the VE. This result is highlighted in Figure 4B, where there are two boxplots showing the average z-score distributions, calculated on the interval *t*_*m*_, for both High and Low Presence groups. The Student's *t*-test resulted in a significant increase of HR during the observation of VEs perceived with high presence (*t* = 2.908, *p* = 0.007).

Analysis performed on the SCL returned no significant difference (*t* = −1.170, *p* = 0.252).

**Figure 5:**
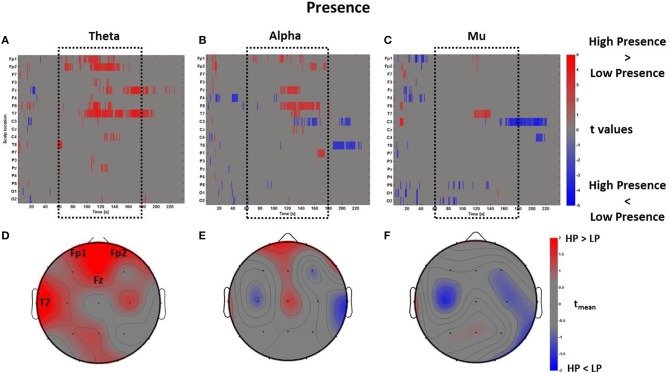
**Time-frequency patterns of PSD for Presence**. **(A–C)** Raster diagram showing in red (blue) significant increase (decrease) of *t*-values for the EEG activity related to the perception of VEs with High Presence (Low Presence) in theta **(A)**, alpha **(B)**, and mu **(C)** bands. Gray color indicates no statistically significant difference (*p* < 0.05, FDR corrected). The dotted black box indicates the time window with most of activations. **(D–F)** Scalp topographic map of average *t*-values for theta **(D)**, alpha **(E)**, and mu **(F)** bands computed in the time window *t*_*m*_ of VE experience. Black labels indicate the scalp sites which show *t*_mean_ > 2.

The *PSDw* datasets were contrasted by computing multiple *t*-test (*p* < 0.05, FDR corrected). Such calculation was performed separately for each frequency band of interest. In Figure 5 are represented the results of the mass univariate analysis in the theta band that was used to inspect cerebral areas involved in assessing the level of presence (Sanchez-Vives and Slater, 2005; Slobounov et al., 2015).

In Figure 5A the statistically significant increase of activation related to High Presence condition is highlighted in red, while the color blue represents Low Presence. This result shows that VEs perceived with high presence elicited a larger amount of theta power across frontal (Fp1, Fp2, Fz) and left temporal (T7) scalp sites, which was mainly found during the central part of the VE experience. This evidence is summarized in Figure 5B, which shows an average topographic map of *t*-values computed within the time window *t*_*m*_. Again, red color highlights an increase of theta activity across frontal and left temporal locations (*t*_mean_ > 2). The analysis of alpha and mu band revealed a few seconds of significant activations at frontal and central sites (Figures 5B,C) which, on average, did not result in a sustained activity (Figures 5E,F).

### PSD time-frequency patterns of perceptual dimensions

A similar analysis was performed to contrast the perceptual dimensions of Familiarity, Comfort, Pleasantness, Arousal, and Novelty.

**Figure 6:**
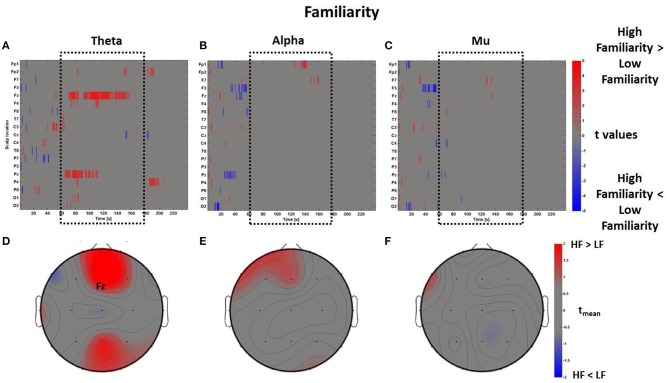
**Time-frequency patterns of PSD for Familiarity**. **(A–C)** Raster diagram showing in red (blue) significant increase (decrease) of *t*-values for the EEG activity related to the perception of VEs with High Familiarity (Low Familiarity) in theta **(A)**, alpha **(B)**, and mu **(C)** bands. Gray color indicates no statistically significant difference (*p* < 0.05, FDR corrected). The dotted black box indicates the time window related to the middle part of VE experience. **(D–F)** Scalp topographic maps of average *t*-values for the theta **(D)**, alpha **(E)**, and mu **(F)** bands computed in the time window *t*_*m*_ of VE experience. Black labels indicate the scalp sites which show |*t*_mean_|> 2.

The perceptual z-scores of Familiarity were divided into positive and negative scores to compare the *PSDw* between High Familiarity condition (z-scores > 0, 17 observations) with Low Familiarity condition (z-scores < 0, 18 observations), respectively. The results of these comparisons are shown in Figure 6 in which raster diagrams depict the significant increase of spectral activity in the different bands of interest, second by second, as investigated by the mass univariate analysis. In this case, in the middle time window *t*_*m*_ (i.e., the central part of the VE perception as highlighted by the dotted black box) there is an increase of theta activity at electrodes Fz and Pz, related to High Familiarity condition (Figure 6A). This pattern of activation is also visible through a topographic map which shows mainly the frontal rhythm with *t*_mean_ > 2 (Figure 6D). The raster diagrams of the alpha (Figures 6B,E) and mu (Figures 6C,F) bands do not provide any particularly sustained activation in the Familiarity condition.

**Figure 7:**
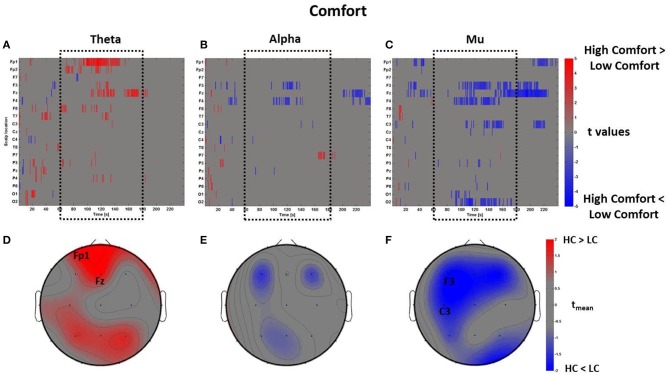
**Time-frequency patterns of PSD for Comfort**. **(A–C)** Raster diagram showing in red (blue) significant increase (decrease) of *t*-values for the EEG activity related to the perception of VEs with High Comfort (Low Comfort) in theta **(A)**, alpha **(B)**, and mu **(C)** bands. Gray color indicates no statistically significant difference (*p* < 0.05, FDR corrected). The dotted black box indicates the time window related to the middle part of VE experience. **(D–F)** Scalp topographic maps of average *t*-values for the theta **(D)**, alpha **(E)**, and mu **(F)** bands computed in the time window *t*_*m*_ of VE experience. Black labels indicate the scalp sites which show |*t*_mean_|> 2.

The mass univariate analysis was then performed for the perceptual dimension of Comfort. As described above, in this condition the perceptual z-scores of Comfort were used to divide the *PSDw* dataset into two groups: High Comfort (z-scores > 0, 19 observations) and Low Comfort (z-scores < 0, 13 observations) undergoing the multiple *t*-tests. The results are shown in Figure 7, which depicts the raster diagrams for the bands of interest (Figures 7A–C), as well as their corresponding average topographic maps (Figures 7D–F). An increase of theta activity across the frontal midline (Fz and Fp1) associated to the High Comfort condition is visible from the raster diagram and the average scalp map within the time-window *t*_*m*_. Although the alpha band shows significant de-synchronization for the same condition across frontal sites, the average map computed within the interval *t*_*m*_ does not reveal any locations with |*t*_mean_|> 2. Instead, the analysis computed in the mu band returned significant de-synchronization across left central (C3) and frontal (F3) electrodes, as visible by the scalp map presenting |*t*_mean_|> 2.

**Figure 8:**
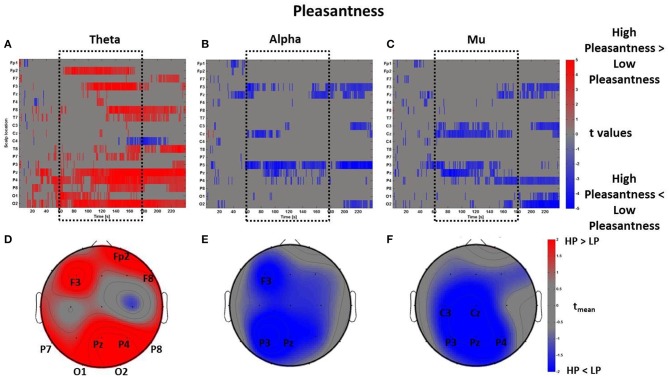
**Time-frequency patterns of PSD for Pleasantness**. **(A–C)** Raster diagram showing in red (blue) significant increase (decrease) of *t*-values for the theta activity related to the perception of VEs with High Pleasantness (Low Pleasantness) in theta **(A)**, alpha **(B)**, and mu **(C)** bands. Gray color indicates no statistically significant difference (*p* < 0.05, FDR corrected). The dotted black box indicates the time window related to the middle part of VE experience. **(D–F)** Scalp topographic maps of average *t*-values for the theta **(D)**, alpha **(E)**, and mu **(F)** bands computed in the time window *t*_*m*_ of VE experience. Black labels indicate the scalp sites which show |*t*_mean_|> 2.

By using the perceptual z-scores of Pleasantness, the *PSDw* dataset was divided into two groups associated with High Pleasantness (z-scores > 0, 22 observations) and Low Pleasantness (z-scores < 0, 13 observations) respectively. The results of the mass univariate analysis are reported in Figure 8, Figures 8A–C show the raster diagrams of the mass univariate analysis, while Figures 8D–F depict the average topographic map of *t*-values within the time window *t*_*m*_ of VEs perception. Figure 8A emphasizes a wide increase of theta activity across occipito-parietal (P7, Pz, P4, P8, O1, O2) and frontal (F3, Fp2, F8) areas for the High Pleasantness condition. Instead, Figures 8B,C show the de-synchronization of the alpha band related to left frontal (F3) and parietal areas (P3, Pz) as well as a wide suppression of the mu rhythm across parietal (P3, Pz, P4) and left central (C3, Cz) regions. For the Pleasantness condition, cerebral activations exceeded the central part of the VE experience and seemed to accompany the subject until the end of the VEs experience. These results are also visible in the average topographic maps (Figures 8D,E).

**Figure 9:**
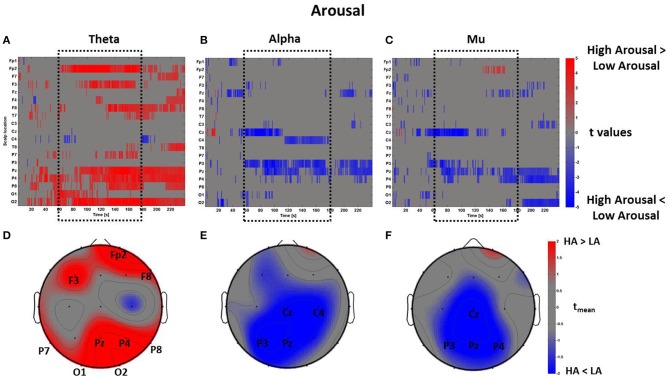
**Time-frequency patterns of PSD for Arousal**. **(A–C)** Raster diagram showing in red (blue) significant increase (decrease) of *t*-values for the theta activity related to the perception of VEs with High Arousal (Low Arousal) in theta **(A)**, alpha **(B)**, and mu **(C)** bands. Gray color indicates no statistically significant difference (*p* < 0.05, FDR corrected). The dotted black box indicates the time window related to the middle part of VE experience. **(D–F)** Scalp topographic maps of average *t*-values for the theta **(D)**, alpha **(E)**, and mu **(F)** bands computed in the time window *t*_*m*_ of VE experience. Black labels indicate the scalp sites which show |*t*_mean_|> 2.

The same mass univariate analysis and average topographic maps were performed for the perceptual dimensions of Arousal and Novelty. The results for Arousal are highly correlated with the ones regarding Pleasantness, as visible in Figure 9. In fact, the Pearson's coefficients were calculated between the distributions of average *t*-values within the time window *t*_*m*_ summarizing the spectral activity of these two dimensions. The results show that the average *PSDw* of Arousal and Pleasantness are positively correlated in all bands (theta: *R* = 0.93, *p* < 0.01; alpha: *R* = 0.94, *p* < 0.01; mu: *R* = 0.90, *p* < 0.01. Bonferroni corrected).

**Figure 10:**
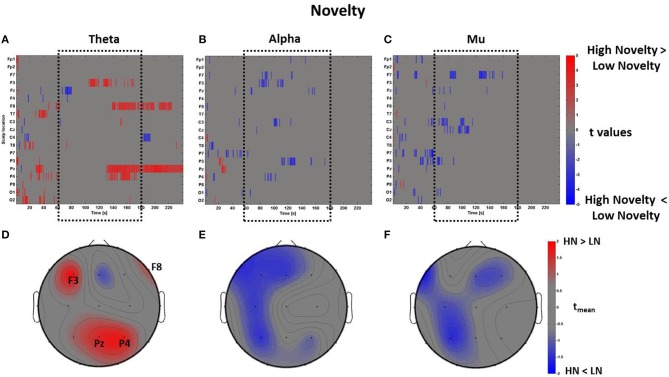
**Time-frequency patterns of PSD for Novelty**. **(A–C)** Raster diagram showing in red (blue) significant increase (decrease) of *t*-values for the theta activity related to the perception of VEs with High Novelty (Low Novelty) in theta **(A)**, alpha **(B)**, and mu **(C)** bands. Gray color indicates no statistically significant difference (*p* < 0.05, FDR corrected). The dotted black box indicates the time window related to the middle part of VE experience. **(D–F)** Scalp topographic maps of average *t*-values for the theta **(D)**, alpha **(E)**, and mu **(F)** bands computed in the time window *t*_*m*_ of VE experience. Black labels indicate the scalp sites which show |*t*_mean_|> 2.

In line with this result, we report another positive correlation between the spectral values related to the perceptual dimensions of Pleasantness and Novelty specifically in theta (*R* = 0.76, *p* < 0.01) and alpha (*R* = 0.68, *p* < 0.01) bands, even though the average topographical t maps of Novelty did not reveal average values of |*t*_mean_|> 2 (Figure 10).

## Discussion

In this study, the electroencephalographic activation underpinning the perception of VEs with different architectural appearances were investigated testing the hypothesis that variations in the virtually presented interiors could activate different cerebral circuits involved in mechanisms of embodiment. For this purpose, the EEG and autonomic activity were recorded during the visual exploration of three VEs. Each environment was designed with the intention to elicit different opinions on the perceptual dimensions of Pleasantness, Novelty, Familiarity, Comfort, and Arousal. Concretely, each perceptual dimension was investigated by contrasting the cerebral activity related to the visual exploration of VEs with higher scores against the one of the VEs with lower scores. In order to select the most relevant time window of exposition, the neurophysiological data regarding the dimension of Presence were analyzed, highlighting the cerebral areas involved during this phenomenon and its specific temporal interval of interest.

Specifically, VEs judged with higher Presence scores revealed the involvement of frontal midline theta power. Similar activations were also found during the perception of familiar and comfortable VEs. Statistical comparisons related to the perceptual dimension of Pleasantness returned a complex pattern of activation in the analyzed bands. In particular, the theta band was characterized by a spread enhancement of activity across occipito-parietal and frontal networks, whereas the alpha band returned a de-synchronization of left parietal and frontal sites. Finally, the perception of both highly pleasant and comfortable VEs showed a de-synchronization of the mu rhythm mostly located in the left hemisphere. A detailed discussion of the aforementioned results is presented in the following sections.

### Sense of presence and sensorimotor integration

IVR is a powerful tool for the investigation of the complex human behaviors during the natural interaction with the external world due to its capacity to represent real-life events and situations in highly controlled computer-generated environments (Tarr and Warren, 2002; Sanchez-Vives and Slater, 2005; Bohil et al., 2011; Dombeck and Reiser, 2012). The experience of IVR can elicit the illusory sensation of being physically present in the VE, this sensation is defined as sense of presence (Slater and Wilbur, 1997; Witmer and Singer, 1998; Diemer et al., 2015). The sense of presence leads to behavioral and neurophysiological reactions corresponding to real-life experience (Slater, 1999; Sanchez-Vives and Slater, 2005; Parsons and Rizzo, 2008). The intensity of these reactions depends on the number and range of the participant's sensory and motor channels connected to the virtual environment through different technological devices (Slater, 1999). For this purpose the VEs were recreated using a CAVE system which provides a high degree of sensorial immersion in the virtual world (Cruz-Neira et al., 1993).

The data show that the designed VEs were able to induce various degrees of presence, as revealed by the adopted questionnaire (Sanchez-Vives and Slater, 2005; Parsons and Rizzo, 2008; Figure 3A). Evidence led us to assess the cerebral pattern and autonomic activation underpinning the sense of presence and, accordingly, to consider the time interval characterized by the increase of frontal midline theta activity as the most significant for the tested architectural experience.

Other studies also report significant correlations between presence and emotion (Baños et al., 2008), suggesting that a higher sense of presence could favor perception of emotional states. Similarly, physiological studies on the autonomous system report an increase of heart rate as a quantitative measure of Presence (Meehan et al., 2005), an observation which was also found in the present study. Based on the EEG data analysis, the cerebral areas which were mostly involved in the evaluation of presence were the frontal and orbitofrontal areas as well as the left temporal region. Specifically, an increase of theta activity was reported across these sites during the perception of VEs rated with High Presence scores. Similar results were also reported by two recent works on spatial navigation tasks using virtual reality. In particular, Slobounov and colleagues illustrate that during the state of presence in immersive 3D scenario subjects showed an enhancement of frontal midline theta (FM-theta) correlated with the success rate in a spatial navigation task, especially during the route encoding. This theta activity was considered to be a reflection of action monitoring, cognitive control and learning (Slobounov et al., 2015). Similarly, Kober and Neuper (2011) report an increase of FM-theta during the processing of familiar spatial cues. Both studies support the sensorimotor integration hypothesis which assigns to theta oscillations the role of coordinating the sensory information with a motor plan to direct wayfinding behavior toward known goal locations (Bland and Oddie, 2001; Caplan et al., 2003). Other studies also report a correlation of FM-theta with hippocampal theta activity during spatial navigation tasks in VEs (Caplan et al., 2003; Ekstrom et al., 2005), although the connection between theta cortical activity and hippocampus is still questioned (Mitchell et al., 2008).

Fronto-central theta rhythms are also increased during meditation and states of internalized attention (Aftanas and Golocheikine, 2001; Baijal and Srinivasan, 2009). Furthermore, theta band power increases with task demands and could be related to orienting (Dietl et al., 1999), memory (Klimesch, 1999), and affective processing (Sammler et al., 2007; Vecchiato et al., 2011a). This cerebral feature also appears during the state of concentration in mental and meditative tasks reflecting focused attentional processing and could be correlated with autonomous activity (Kubota et al., 2001). Therefore, the sense of presence could elicit mechanisms underlying sensorimotor integration as well as cerebral networks regulating focused attention, as reported in this study.

In addition, FM-theta was not only elicited during the experience of presence but also during the visuospatial exploration of more familiar, comfortable and pleasant environments. These findings may reflect recruitment of theta oscillations in focused attention, memory and positive emotional experience mechanisms associated with the exploration of VEs. Therefore, the recognition of familiar features in the environment, as well as the perception of comfort, could activate those cerebral circuits involved in internalized attention, relaxation and hence favor sensorimotor integration in space.

Overall, these results show that the discussed cerebral activations are common to the dimensions of Presence, Familiarity and Comfort, all showing an increase of theta frontal activity. However, an additional region was characterized by Presence only, which is the left temporal site. In their review, Jäncke and colleagues report the activation of a wide fronto-parietal network when participants reported a strong sense of presence in a virtual roller coaster scenario (Jäncke et al., 2009). Interestingly, these authors reported a difference between patterns of effective connectivity in adolescents and children. They attributed this finding to the prefrontal cortex which is not fully matured in children. Also, children are able to engage multi-sensory integration areas such as the temporo-parietal junction (TPJ) which is known to be a key area for studying self-location, i.e., the ability to place and experience oneself in the physical space (Aglioti and Candidi, 2011). TPJ is a cerebral region processing the body space proprioception and bodily awareness integrating signals coming from the body. This cortical area is involved in mental tasks where self-relocation is required, such as transcending body-related sensorimotor experiences usually occurring during meditative states (Urgesi et al., 2010). In particular, Ionta and colleagues reveal that multisensory integration in TPJ reflects the feeling of being an entity localized at a position in space, allowing the perception of the world from this perspective (Ionta et al., 2011).

An additional interpretation for this finding could be to connect the activation of the left temporal lobe with functions of visuomotor coordination and motor representation. Tankus and Fried (Tankus and Fried, 2012) discovered two neural populations in the human temporal-lobe activated differently during a motor and a visuomotor task, respectively. The second group of neurons, connected with the parahippocampal gyrus, had already been demonstrated to respond to visual motion (Sato and Nakamura, 2003) as well as to the observation of paintings reproducing landscapes (Kawabata and Zeki, 2004; Yue et al., 2007). It is known that images depicting environments activate the parahippocampal place area (PPA), an association area which codes place-related information and mediates contextual association with the environment (Epstein and Kanwisher, 1998; Bar, 2004). In fact, this area might not have been elicited in the above mentioned virtual roller coaster experiment because it was made of non-static movement scenes (Jäncke et al., 2009). Hence, these results seem to support the view that PPA could play a role in the cerebral circuit of presence.

### Aesthetic experience

The pattern of cerebral activations related to High Pleasantness returned increased EEG power in all the investigated frequency bands. Specifically, the perception of High Pleasantness environments activated not only mechanisms related to action planning but also to the sensory visual areas and frontal regions. According to a recent theoretical and experimental framework, aesthetic experience may involve the interaction of a neural systems' triad formed by the sensory-motor, the emotion-evaluation and the meaning-knowledge circuits (Chatterjee and Vartanian, 2014). Therefore, this perspective unifies both the sensory (e.g., pleasantness is positively correlated with the activation of sensory areas; Zeki, 1999) and the conceptual (e.g., pleasantness is positively correlated with the activation of frontal regions mediating concepts; Cela-Conde et al., 2004) hypotheses. In this regard, a recent study conducted by Thakral and colleagues show the activation of both visual and frontal cortices thus supporting sensory and conceptual hypotheses of aesthetic experience (Thakral et al., 2012). More specifically, these authors disentangle the response of the visual areas elicited by the observation of motion pictures from the frontal activity elicited by the observation of pleasant images. Their results provide evidence that motion experience is associated with activity in motion processing regions, while the experience of pleasantness is associated with the anterior prefrontal cortex. However, these two processes seem to interact in the same window of activation to engender the aesthetic experience (Lorteije et al., 2006).

Similarly, the results obtained in this study show an increase in the theta band in occipital, frontal and orbito-frontal regions during the perception of highly pleasant VEs. Figure 3 illustrates that the furnished virtual rooms account for the “High Pleasantness” condition while the empty virtual room was evaluated as “Low Pleasantness.” Accordingly, it might be reasonable to argue that the activity of the cerebral areas accounting for the amount of potential motion is generated by the possibility to interact and move around the objects located inside the virtual rooms. The activation of occipital areas had already been shown through the comparison of pictures with implied motion against the ones with non-implied motion (Lorteije et al., 2006). Other paradigms investigating the multisensory perception of objects in motion (Senkowski et al., 2007) and the processing of coherent meaningful objects (Vanni et al., 1997) have achieved the same results.

At the same time, the activity of the frontal lobe during perception of pleasantness is caused by the conceptual content of the stimulation as already reported in research papers (Cela-Conde et al., 2004; Ishizu and Zeki, 2011), meta-analysis (Kühn and Gallinat, 2012), and review (Chatterjee and Vartanian, 2014).

The activity of the parietal cortex, as indicated by the present results, could reflect the integration of multisensory information from different sensory modalities to form a coherent multimodal representation of space, which is coded in a body-centered reference frame (evidence already reported in a VR study, Jäncke et al., 2009). The integration of multisensory cues around the body in the peripersonal space serves to map the position of objects in the surrounding environment in terms of one's own body. In addition, visual targets elicit a motor schema for potential action that maps the position of objects in the surrounding environment, irrespective of whether the corresponding action is actually executed (Jeannerod et al., 1995; Rizzolatti et al., 1997a,b). In their virtual roller coaster scenario, Jäncke et al. (2009) argue that VEs trigger motor schemas mapping the visual objects in terms of real motor space as well as a corresponding plan for potential action.

Another study reports that the activity of the parietal cortex could be also modulated by the object's size (Tarantino et al., 2014), while other authors discuss that parietal areas are involved in integrating information about three-dimensional objects, such as the object size and the grasp-relevant dimension (Monaco et al., 2014). In addition, Salmi et al. (2014) illustrate that the parietal activity related to goal-directed actions could also depend on a behavioral priority accounting for percepts, thoughts and emotions during the observation of natural scenes.

In this regard, the presented results show a theta increase across the right parietal cortex during the perception of pleasant VEs. In agreement with the aforementioned literature, these findings could offer the interpretation that the perceived pleasant VEs may favor the triggering of motor schemas related to potential actions planning.

The perception of pleasant VEs also returned a significant activation of the alpha band in left-central parietal and frontal areas, a fact which might underlie an increase in visuospatial processing. In fact, similar findings were already reported in a study performed with fMRI, which tested the level of pleasantness during the observation of spaces with varying architectural features (ceiling height and openness/enclosure), illustrating the activation of left precuneus and left middle gyrus (Vartanian et al., 2015). These two structures have an important role in visuospatial processing (Kravitz et al., 2011). Other authors argue that this lateralized activation of the left hemisphere could be due to the processing of categorical spatial relations and not to the processing of coordinate spatial relations (Amorapanth et al., 2009). Categorical spatial relations are involved in tasks that do not require a precise location. On the other hand, coordinate spatial relations require precise metrical information about distances among objects (Kosslyn, 1987).

Additional findings report that the left hemisphere is more involved in processing spatial relations, while the activity of the right parietal lobe relates to coordinate spatial relations (Baciu et al., 1999; Suegami and Laeng, 2013). Interestingly, Cela-Conde et al. (2009) show that in women there is a lateralized activity in the left parietal areas during the observation of stimuli rated as beautiful. Asymmetrically, men show an increase of parietal activity in the right hemisphere. Taking an evolutionary perspective, they discuss that women rely more on categorical strategies when processing objects more than men do. Due to the lack of statistical power, evidence supporting this gender differentiation cannot be provided in this study and should be further investigated.

However, since the visual exploration of VEs did not require the processing of specific distance among objects, it is reasonable to think that subjects mostly activated categorical spatial processing during the perception of pleasant environments. This kind of processing could be related to the fact that people were considering the quality of the space as a whole. Vartanian et al. (2015) report a positive linear correlation between the activation of the left precuneus and the scores of pleasantness. Similarly the presented results show a potential role of the left precuneus in the perception of pleasant VEs, an area which also facilitates visuospatial exploration. This finding could be also interpreted according to the biophilic hypothesis (Kellert and Wilson, 1995; McVay et al., 1995), which suggests that the activations of areas for visuospatial exploration could support more general human preferences for appreciating spatial properties that are evolutionary beneficial.

### Embodiment and affordance

The data indicate a certain degree of similarity in the pattern returned by the mu band pertaining to the perceptual dimensions of Pleasantness and Comfort. In fact, both dimensions highlighted a de-synchronization of such rhythm during the perception of VEs with High Comfort and High Pleasantness scores. Along with several studies showing a primary importance of cognition for art response (Cinzia and Vittorio, 2009; Chatterjee and Vartanian, 2014), recent theories and experimental works propose that the activation of embodied mechanisms play a key role in the aesthetic experience of works of art and that these mechanisms could also account for the perception of architectural spaces (Freedberg and Gallese, 2007; Umilta' et al., 2012; Sbriscia-Fioretti et al., 2013). In summary, the motor system is activated due to an automatic empathic relationship established between the artwork and the observer—a phenomenon which could be triggered by the work's representational content and by artist's creative gestures (Cinzia and Vittorio, 2009). Umilta' et al. (2012) report the suppression of the mu rhythm, recorded around electrodes C3 and C4, during the observation of the cuts on the canvases by Lucio Fontana. Similarly, Sbriscia-Fioretti et al. (2013) illustrate that observing the brushstrokes by Franz Kline engages motor areas along with the occipital circuits related to vision, as well as the frontal and orbitofrontal regions processing reward and judgment, respectively. In both studies, the authors contrasted the EEG activity gathered from the perception of the original works against the activity elicited by the observation of computer-generated reproductions displaying the same patterns of lines and stripes but without the original dynamic components (i.e., artist's gestures). Thus, the described cerebral activations are engendered by the automatic comprehension of those dynamic components and the recognition of the motor actions that produced those works. Moreover, the activity in premotor and motor cortices has been observed in other tasks involving spatial cognition (Rizzolatti and Craighero, 2004). In this study, the perception of VEs rated as pleasant and comfortable could involve spatial cognitive processes, increased somatosensory perception and the planning and execution of movements. In this sense, subjects felt free to “live” those spaces which triggered the embodied mechanism (Freedberg and Gallese, 2007; Cela-Conde et al., 2009).

Similarly, object perception provides an example of embodiment which resides in the action domain. Specifically, the observation of manipulable objects triggers the same motor resources typically employed during the planning and execution of actions targeting the same objects (Gallese and Sinigaglia, 2013). Hence, the motor system can also be engaged in the absence of active action execution. Recent studies show that observing manipulable objects can lead to mu rhythm suppression within 300 ms after stimulus presentation, possibly reflecting the automatic access to object-associated actions (Proverbio, 2012; Rüther et al., 2014). In particular, Proverbio shows that this activation is evident when comparing the observation of manipulable objects (tools) to the observation of non-manipulable objects, mostly in the band between 10 and 12 Hz (Proverbio, 2012). In a similar fashion, Rüther and colleagues report a significant suppression of the mu rhythm when observing familiar tools instead of new ones, although in a band ranging in lower frequencies (i.e., from 8 to 10 Hz; Rüther et al., 2014). Accordingly, during the perception of VEs, the modulation of the mu rhythm seems to depend not only on the simple observation of specific objects in the environment but also on the perception of the environment as a whole. In fact, the analysis regarding Pleasantness essentially compares the VEs with objects against VEs without objects (Figure 3E). On the other hand, when analyzing Comfort, the High Comfort condition had both VEs with and without objects, making the comparison with the Low Comfort condition object-balanced (Figure 3D). In other words, both the High (*z* > 0) and Low (*z* < 0) Comfort datasets comprise the perception of VEs with and without objects. Therefore, these results show the suppression of the mu rhythm in the conditions of High Pleasantness and High Comfort, which could reflect the possibility to interact with the objects located in the VEs.

Because the objects were perceived in a specific functional configuration instead of being observed independently, this neurophysiological mechanism could have a role in regulating potential actions which influence the pleasantness and comfort of the environment as a whole. These results are in line with other studies performed on object affordances, showing that the functional identity of graspable objects influences the extent to which they are associated with motor representations (Creem-Regehr and Lee, 2005; Proverbio, 2012). Similarly, the object familiarity could enhance the activation of action representations and motor plans (Rüther et al., 2014). Such findings reveal that the view of a tool automatically activates appropriate motoric properties, including its affordance and the representation of the associated motor interaction. In addition, behavioral and brain stimulation studies have also shown that the affordability is context dependent and that spatial constraints affect one's reuse of his/her own action representations (Costantini et al., 2010, 2011; Cardellicchio et al., 2011).

Finally, the analysis of Familiarity and Novelty returned no significant results in the mu band, probably because the EEG activity in both the “high” and “low” groups accounted for the perception of objects with the same function (i.e., affordance) but located in different VEs, and with different design. This can be observed in the distribution of the related behavioral z-scores in Figures 3B,C. However, further investigation is needed in order to explore how the perception of affordances depends on the architectural context. This would elucidate the relationship between embodied mechanisms and the specific features of architectural environments.

### Limitations

Due to the explorative nature of the present study, several limitations should be taken into account when considering the final results. First, the tested VEs were designed with the aim to induce different levels of Familiarity, Novelty, Comfort, Pleasantness, and Arousal. Therefore, the corresponding main cerebral activations were investigated regardless of the specific features of the spaces represented (e.g., VE1 vs. VE2). The aim was to test a simple IVR setup and at the same time to effectively retrieve neurophysiological correlates of environment perception. These results could be useful for shaping architectural hypotheses in future studies. Secondly, the electromiographic activity of the subjects enrolled in the data recording was not controlled, still they were asked to seat in the CAVE without moving their legs, arms and hands. During the data collection, the experimenter monitored the behavior of all subjects during the task and did not report any movement of their limbs. Hence, the activity of the motor areas could have been caused only by cerebral processes which are not related with movement execution. Also, the gaze was not controlled using eye-tracking measurements and therefore the objects and locations of the VEs that were the most looked at by the participants could not be defined. Instead, the aim was to retrieve neurophysiological correlates of a generalized perception—that is, of architectural space as a whole and not of the specific visual targets in the environment. Finally, from the architectural point of view, the setup required the participants to explore the VEs with a certain degree of attention. Conversely, in everyday life, people usually do not focus on architectural features but rather live the space in a habitual and automatic manner, without paying special attention to the details of the environment they are experiencing. Due to the correlational design of this work, all the points mentioned above will be addressed with additional control conditions to improve the setup of further experiments.

## Conclusions

The present findings aim to provide new insights for studying the impact of architecture on human brain. These results revealed that perception of familiar and comfortable real-like VEs engender the activation of those cerebral circuits elicited by the sense of presence which facilitate sensorimotor integration. Similarly, the perception of pleasant environments involves areas devoted to visuospatial processing, suggesting the importance of a fronto-parietal network in aesthetic perception of places. These cerebral areas could be considered as evolutionary beneficial. Finally, a common suppression of the mu rhythm over the left motor areas was reported, characterizing highly pleasant and comfortable environments. These results are in agreement with the embodied simulation theory, which plays a fundamental role in object perception and possibly in the environment perception as a whole. Overall, this study shows the involvement of motor and cognitive processes for the evaluation of architectural environments. Further research is needed for in-depth investigation of the role of embodiment, affordances and perceptual processes underpinning the appreciation of architectural environments. This knowledge will provide neurophysiological findings to improve the design of buildings and help to create environments that satisfy man's demands.

### Conflict of interest statement

The authors declare that the research was conducted in the absence of any commercial or financial relationships that could be construed as a potential conflict of interest.
